# Rheumatology around the world: perspectives from Australia and New Zealand

**DOI:** 10.1186/s13075-017-1246-8

**Published:** 2017-02-10

**Authors:** Fiona M. F. McQueen

**Affiliations:** Department of Molecular Medicine and Pathology, Faculty of Medical and Health Sciences, University of Auckland, 85 Park Rd., Grafton, Auckland, New Zealand

## Abstract

Rheumatology continues to be an exciting and vibrant specialty for specialists practising in New Zealand and Australia. Clinicians follow treat-to-target regimens to manage peripheral and axial inflammatory arthritides using conventional and biological agents, which have revolutionised management of rheumatic disease over the past two decades. However, optimal clinical practice has significant pharmacoeconomic implications which impact on health funding at a national level, and the advent of biosimilars is keenly awaited. The management of non-inflammatory rheumatic disease and the lack of effective disease-suppressing pharmacologic therapy for osteoarthritis continue to challenge clinicians. We are fortunate in having world-class rheumatology research in our region with basic scientists and clinical rheumatologists spearheading investigations, the ultimate aim of which is to improve the quality of life for our patients.

## Background

Rheumatology continues to be an exciting and vibrant specialty for specialists practising in New Zealand and Australia. There have been important advances on the research front over the past 12 months as well as ongoing improvements in clinical practice. Challenges in our region are the same as those faced by rheumatologists world-wide, namely to gain access to the potent biological agents now available for suppression of joint inflammation and damage in rheumatoid arthritis (RA), spondyloarthropathies (SpA) and other inflammatory rheumatic diseases for those patients who do not respond to conventional therapies, while trying to prevent or minimise adverse effects.

## Clinical challenges across the region

Clinicians continue to follow treat-to-target regimens according to international guidelines that utilise outcome measures such as the Disease Activity Score 28 (DAS28) in RA and the Bath Ankylosing Spondylitis Disease Activity Index (BASDAI) in SpA to ensure therapy is escalated to attain optimal clinical responses. At the same time, there are ongoing challenges in managing chronic pain and non-inflammatory rheumatic conditions such as fibromyalgia where there have been fewer advances in diagnosis and management. Similarly, a lack of effective disease-suppressing agents for use in osteoarthritis (OA) is frustrating, and the rheumatologist is frequently left out of the clinical care loop altogether due to funding constraints with suboptimal outcomes for many patients. Another level of complexity has been added as health budgets blow out with escalating pharmaceutical costs, so that pharmacoeconomic factors increasingly impact upon clinical decision making. A wide range of anti-cytokine and disease-modifying biological therapies including those blocking the actions of tumour necrosis factor (TNF)α, interleukin (IL)-6, IL-17, IL-1, and costimulatory molecules, plus B-cell depleting agents, are now publically funded in Australia for the management of inflammatory arthritides. The most recent addition is the oral Janus kinase (JAK) inhibitor, tofacitinib, which was registered for use by the Pharmaceutical Benefits Scheme (PBS) in October 2015. Only a few months later, the first biosimilar (Inflectra) was also listed. The Australian government is investing $20 million over 2015–2018 to improve awareness and confidence in biosimilar medicines for both health professionals and consumers [1]. A parallel situation is evolving in New Zealand, which is strongly influenced by Pharmaceutical Management Agency (PHARMAC) policy. This organisation has been extraordinarily effective in juggling medical and business priorities to obtain the best deal for biologics in many areas of medicine, aiming to ensure access and affordability for a nation of 4.5 million people with a small taxpayer base. In 2015, adalimumab was the highest cost single item listed, doubtless influenced by the relatively restricted number of anti-TNF agents available in New Zealand (adalimumab, etanercept, and infliximab only), but highlighting the cost impact of anti-rheumatic drugs at a national level [2].

## Australian initiatives in RA, systemic sclerosis, and osteoarthritis

Australian research initiatives continue to cover a broad range of conditions. Professor Ranjeny Thomas and her group at the University of Queensland are pursuing exciting work that aims to develop a vaccine for RA. The rationale behind tolerising dendritic cell (DC) immunotherapy has been summarized recently [3], and results from a phase I clinical trial were reported in the past 12 months [4]. “Rheumavax”, comprising autologous DCs that have been modified with a nuclear factor (NF)-kB inhibitor and exposed to citrullinated peptide antigens, was given as a single intradermal injection to anti-citrullinated peptide antigen-positive (ACPA+) RA patients with human leucocyte antigen shared epitope (HLA-SE) risk alleles. Biologically significant T-cell effects were observed, and disease activity was reduced in treated patients within a month. Further studies are planned to better define long-term effects. The role of granulocyte-macrophage colony stimulating factor (GM-CSF) in rheumatic inflammatory disease has been a focus of Australian research for two decades, as recently summarized by Wicks and Roberts [5]. Studies in an experimental arthritis mouse model revealed that combined anti-IL-17 and anti-GM-CSF therapy ameliorated arthritis progression. Thus, this combination might be useful for RA patients who do not fully respond to inhibition of the separate cytokines [6].

The importance of B cells and autoantibody formation in the pathogenesis of systemic sclerosis has recently been summarized [7]. The Scleroderma Consortium based in South Australia continues to produce high quality original cohort-based research that explores links between clinical features and immune markers in this complex disease. A personalized medicine approach using principal components analysis was used in a recent study exploring the significance of an extended autoantibody profile in a cohort of scleroderma patients [8]. Five major autoantibody clusters were identified and associated with clinical subtypes. Moving south to Tasmania, research into osteoarthritis, and in particular the imaging features associated with disease progression, continues at the Menzies Institute for Medical Research, Hobart. Collaboration between Australian and Chinese investigators has found evidence that alterations in the magnetic resonance imaging (MRI) signal intensity of the infrapatellar fat pad (IPFP) predicts increases in ambulatory knee pain and cartilage damage in knee OA [9]. Signal intensity of the IPFP has been proposed as an imaging biomarker for knee OA, and maintaining the size and health of this fat pad may become a priority during total knee arthroplasty to optimize outcome, pending the results of prospective controlled clinical trials [10].

## New Zealand research focusing on gout

New Zealand workers continue to be at the forefront of gout research and have participated in the recent European League Against Rheumatism/American College of Rheumatology (EULAR/ACR) revision of gout classification criteria [11], identification of genetic predisposition markers [12], clarification of environmental triggers, imaging features and identification of optimal approaches to management as summarized recently by Dalbeth et al. [13]. What are some of the important advances? Dual energy computed tomography (DECT) has provided new insights into the nature of this crystal deposition disease by allowing direct visualization of urate deposits in many patients with tophaceous gout and some with asymptomatic hyperuricaemia [14]. The diagnostic role of ultrasound in gout has also recently been studied [15], and this modality was found to have high specificity, even in those with early disease without obvious clinical features. An important environmental trigger is the excessive consumption of sugar-sweetened beverages (SSB), which contain high levels of fructose. As developed countries face skyrocketing levels of obesity and type II diabetes, there has also been an increase in gout, and SSB consumption is likely to be an important precipitant, especially in peoples of Polynesian origin who have inherently raised urate levels due to genetic factors [12]. Unfortunately, a recent study indicates that a high proportion of patients with gout and type II diabetes, including those on haemodialysis, are not responding to health messages to abstain from SSB consumption [16]. Advances in urate-lowering therapy (ULT) include a re-assessment of the place for older drugs such as benzbromarone and allopurinol [13]. A recently completed randomised controlled trial in gout all-comers (including those with renal disease) compared a dose-escalated regimen of allopurinol (up to 600 mg/day) with controls dosed traditionally according to creatinine clearance [17]. Target serum urate (<6 mg/L) was achieved at month 12 in 69% of the dose-escalated participants vs 32% of controls (*p* < 0.001) without added toxicity in this challenging group of gout patients. Febuxostat is another extremely effective agent in this setting and available in New Zealand for patients who have not responded to first-line ULT.

## Conclusions

To summarise, rheumatologists in our region continue to make a major and positive difference to patients’ lives by optimising the use of conventional and biological pharmacologic agents while aiming for a holistic team approach, joining forces with other health professionals and physician colleagues. Research is flourishing and ranges from basic science projects to clinical and epidemiological studies. Investigators in Australia and New Zealand contribute to many multinational collaborative groups whilst also focussing on innovative solutions to local problems.

## Abbreviations

DC: Dendritic cell
GM-CSF: Granulocyte-macrophage colony stimulating factor
IPFP: Infrapatellar fat pad
IL: Interleukin
OA: Osteoarthritis
RA: Rheumatoid arthritis
SpA: Spondyloarthropathies
SSB: Sugar-sweetened beverages
TNF: Tumour necrosis factor
ULT: Urate-lowering therapy
